# Supplementary material for: The causal relationship between the human gut microbiota and pyogenic arthritis: a Mendelian randomization study
Source: Front Cell Infect Microbiol. 2024 Nov 26;14:1452480. doi: 10.3389/fcimb.2024.1452480 (PMC11629706; doi:10.3389/fcimb.2024.1452480)
Supplement: Supplementary file 1 [file DataSheet1.zip › mendelian test/ebi-a-GCST90027832.csv_forest.pdf]

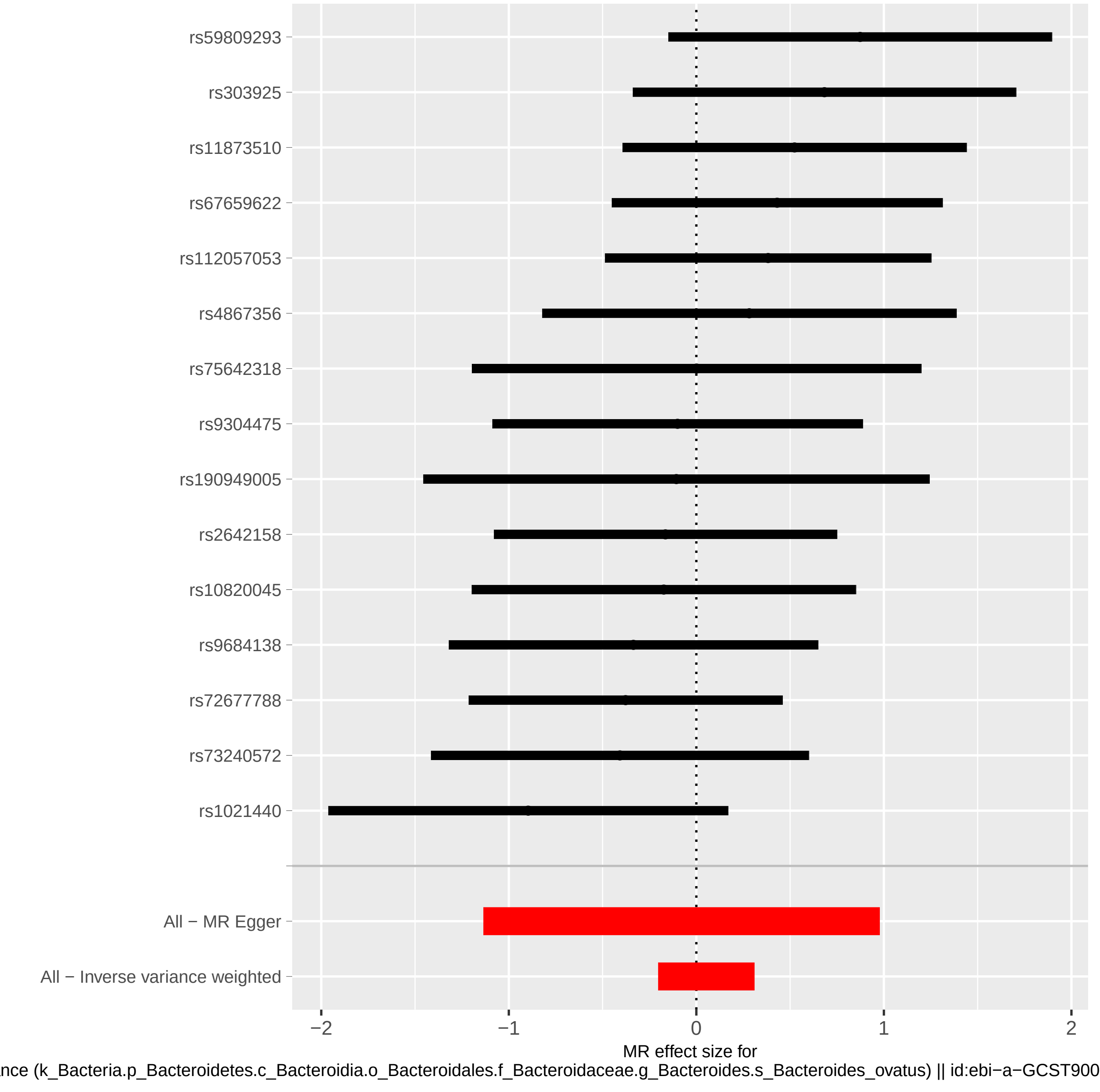
rs59809293
rs303925
rs11873510
rs67659622
rs112057053
rs4867356
rs75642318
rs9304475
rs190949005
rs2642158
rs10820045
rs9684138
rs72677788
rs73240572
rs1021440
All − MR Egger
All − Inverse variance weighted
−2
−1
0
1
2
MR effect size for
nce (k_Bacteria.p_Bacteroidetes.c_Bacteroidia.o_Bacteroidales.f_Bacteroidaceae.g_Bacteroides.s_Bacteroides_ovatus) || id:ebi−a−GCST900
